# Complete Genome Sequences of Seven Strains Composing a Model Bacterial Community of Maize Roots

**DOI:** 10.1128/genomeA.00997-17

**Published:** 2017-09-07

**Authors:** Ben Niu, Roberto Kolter

**Affiliations:** Department of Microbiology and Immunobiology, Harvard Medical School, Boston, Massachusetts, USA

## Abstract

Previously, we assembled a model bacterial community of maize roots. Here, we report the complete genome sequences of the seven strains composing the community.

## GENOME ANNOUNCEMENT

As two key components of the biosphere on our planet, plants and microbes sometimes evolve with each other. Plants serve as a “home” for microbes, one in which they live and interact with other organisms. In return, microbes can affect the growth and health of their plant hosts (1). In a previous study, aided by host selection, we built a model bacterial community for maize root comprising strains belonging to seven species (*Stenotrophomonas maltophilia* AA1, *Ochrobactrum pituitosum* AA2, *Curtobacterium pusillum* AA3, *Enterobacter cloacae* AA4, *Chryseobacterium indologenes* AA5, *Herbaspirillum frisingense* AA6, and *Pseudomonas putida* AA7) (2). This model community is able to colonize maize roots and exhibits a clear protective effect on hosts by suppressing the growth of phytopathogens. Here, we announce the complete genome sequences of the seven strains composing this model bacterial community of maize roots.

The genome sequences of the seven strains were determined by the PacBio single-molecule real-time (SMRT) sequencing technology at the University of Delaware DNA Sequencing and Genotyping Center. The genomic DNA of each strain was extracted from pure cultures grown overnight in tryptic soy broth (BD) using the cetyltrimethylammonium bromide-based extraction protocol (3), including proteinase K and RNase treatments. The SMRTbell template preparation kit (Pacific Biosciences, Menlo Park, CA, USA) was used according to the PacBio standard protocol (20-kb template preparation using the BluePippin size-selection system). After DNA size selection, the SMRT cells were run on a PacBio RS II instrument (Pacific Biosciences) using a P4-C2 chemistry combination. Adaptor trimming, quality filtering, and assembly were performed using the hierarchical genome assembly process pipeline (4). The assembly resulted in 49,930 to 87,018 reads with mean lengths of 10,932 bp to 12,808 bp and *N*_50_ read lengths of 14,762 bp to 17,399 bp for the seven strains.

The genome sequences for five of the strains—* Stenotrophomonas maltophilia* AA1, *Enterobacter  cloacae* AA4, *Chryseobacterium indologenes* AA5, *Herbaspirillum frisingense* AA6, and *Pseudomonas putida* AA7—contain one contig, while those of *Ochrobactrum pituitosum* AA2 and *Curtobacterium  pusillum* AA3 are composed of four and two contigs, respectively. Three of the four contigs of AA2 are chromosomes, while the remaining one is a putative plasmid. The two contigs of AA3 are one chromosome and one putative plasmid, respectively. The genome size of the seven strains ranges from 4,541,048 bp (AA3) to 6,137,024 bp (AA7). The chromosome of AA3 possesses the highest G+C content, 70.87%, while the lowest G+C content is for the genome of AA5, 35.88%. The number of coding sequences ranges from 4,138 (AA1) to 5,490 (AA2). All the genome sequences have been annotated by the Rapid Annotation using Subsystem Technology (RAST) server version 2.0 (5–7). These genome sequences provide us with an overview of the functional genes related to the plant-associated activity of the model bacterial community and should serve as a useful guide and resource for future studies of bacterial interspecies interactions and bacterial community-plant interaction.

### Accession number(s).

This genome sequencing project has been deposited at GenBank under the accession no. CP018756, CP018779 to CP018786, CP018845, and CP018846.
